# Hybrid work and mental distress: a cross-sectional study of 24,763 office workers in the Norwegian public sector

**DOI:** 10.1007/s00420-025-02136-9

**Published:** 2025-03-25

**Authors:** Lorena Edith Trevino Garcia, Jan Olav Christensen

**Affiliations:** 1 Research group for Work Psychology and Physiology, National Institute of Occupational Health, Pb 5330 Majorstuen, 0304 Oslo, Norway; 2 Department of Psychology, University of Oslo, Oslo, Norway

**Keywords:** Mental health, Remote work, Work-family conflict, Flexibility

## Abstract

**Objective:**

Few studies have investigated the relationship between post-pandemic hybrid work-from-home (WFH), mental health, and work-life balance. We examined the association between hybrid WFH, mental distress, availability demands, work-life conflict, and life-work conflict.

**Methods:**

Data from 24,763 office workers in the public sector in Norway were analyzed by linear and logistic regressions.

**Results:**

Employees practicing flexible hybrid WFH (i.e., when needed/desired) were less likely to report mental distress (measured by the Hopkins Symptom-Checklist; HSCL-5) than those not practicing WFH. WFH being self-chosen was associated with less distress. Flexible WFH was also associated with availability demands, work-life conflict, and life-work conflict, which were, in turn, linked to distress. The risk of distress increased with the number of weekly days of flexible WFH. Workers with fixed agreements to regularly WFH did not report significantly less distress than those with no WFH. However, fixed WFH was associated with lower availability demands, not with work-life conflict, and was more often self-chosen than flexible WFH.

**Conclusion:**

Flexible WFH may alleviate distress but may also indicate attempts to cope with taxing availability demands, and may even introduce stressors that could reverse beneficial effects. Our results should motivate nuanced, multifactorial assessments of WFH in organizational practice and research.

**Supplementary Information:**

The online version contains supplementary material available at 10.1007/s00420-025-02136-9.

## Introduction

Rapid societal changes driven by technological advancements, globalization, and disruptive events like the COVID-19 pandemic have triggered a swift adoption of new work practices. A rise in hybrid working from home (WFH) agreements—a variation of remote work where employees work partially from home and partially at the workplace—is one notable change. In Norway, the proportion of workers who had access to hybrid WFH was approximately 4% in 2019, rising to 9.6% in 2022 (STAMI-rapport 2024). This situation suggests a widespread change from traditional settings to flexible working environments, which may allow employees higher job autonomy but potentially also higher availability demands (Knardahl and Christensen 2022). Since demands and autonomy may influence employee mental health, large-scale changes in these factors may have considerable repercussions for occupational health. In Norway, sick leave due to mental health disorders is rapidly increasing. Since 2019, 44% of sickness absences are due to mental health conditions (NAV 2024). Anxiety and depression are among the most common reasons for sickness-related absence and repeated and prolonged sick leave (Knudsen et al. 2013).

Job autonomy, control, and flexibility are terms often used when referring to workers’ agency over their own working agreements and, consequently, their lives (Meyer et al. 2021). Job autonomy influences motivation (Hackman and Oldham 1975), and autonomy is considered a basic human psychological need (Ryan and Deci 2000). Hence, job autonomy, such as having the flexibility to determine when, where, and how to organize one’s work tasks, is a job resource that can promote mental well-being. In addition, it can promote a sense of responsibility and enhance performance (Hackman and Oldham 1975). However, autonomy may also have a “dark side” (Kubicek et al. 2017). For instance, Warr (1994, 2013), with the ‘Vitamin Model’ of environmental factors and mental health, posits that job autonomy can enhance or impair employees’ mental health depending on the extent of it. For example, situations with extremely low or extremely high levels of job autonomy can contribute to declines in psychological well-being (Warr 1994). The former scenario would not give the employee an opportunity to make choices that may improve their situation and promote a sense of agency, whereas the latter could imply a lack of support or assistance in making choices and prioritizing tasks.

Kaduk et al. (2019) found that employees who worked at least 20% of the week from home and reported a strong influence over the choice of place to work were less stressed and had fewer intentions to leave the company. On the other hand, involuntary hybrid work from home (when employees worked during off-work hours at different times and places because the employer required it) was associated with higher levels of work-life conflict, stress, burnout, turnover intentions, and lower job satisfaction. This supports the notion that WFH is not automatically a beneficial working agreement, although employees may value having voluntary, flexible access to hybrid WFH agreements. Hence, it remains pertinent to identify the conditions under which WFH may or may not attenuate mental distress.

Pre-pandemic evidence shows that women found it particularly difficult to balance work and private life while working from home (Eurofound 2020). The double burden hypothesis (Nilsen et al. 2017) suggests that overall, women tend to experience more role strain from combining work and family (Bratberg et al. 2002; Goode 1960). Hence it is important to study male and female workers separately.

Some previous studies found no associations between WFH and work-life balance or motivation (Innstrand et al. 2022), nor between hybrid WFH and self-reported health complaints (Oakman et al. 2022). However, others have found that hybrid WFH may be linked to positive outcomes such as job control (Knardahl and Christensen 2022; Ervasti et al. 2022), organizational commitment (Knardahl and Christensen 2022), improved working environment due to silent spaces that can enhance focus (Bloom et al. 2015), better self-reported health, lower levels of mental distress (Ervasti et al. 2022), and lower sickness absence rates (Mobach and Initiative 2023). Nevertheless, hybrid WFH has also been related to feelings of extended availability demands (Knardahl and Christensen 2022), lower quality of life (Carlsen et al. 2022), and reduced psychological well-being in employees who WFH during the pandemic compared to those who continued working at the office (Nielsen et al. 2022). The mixed evidence regarding the associations between hybrid WFH, job autonomy, and employees' mental well-being could be due to variations among studies regarding conditions under which hybrid WFH is conducive to mental well-being. This problem may be exacerbated by much previous research being based on small samples, which are more sensitive to the influence of contextual moderators. Moreover, small samples limit the possibility of studying heterogeneity in the association between WFH agreements and mental health across different groups (e.g., females and males).

Although organizations in Norway have had to develop regulations regarding access to hybrid WFH (Arbeidstilsynet 2002, 2008), there is still scarce evidence to guide their approaches, and many have to rely on rather improvised and somewhat arbitrary strategies rather than evidence-based approaches. Hence, more evidence is needed to determine the association between hybrid WFH and mental well-being, and this study aims to provide insights into the circumstances under which hybrid WFH benefits employees' well-being. The present study is among the first large-scale post-pandemic studies to investigate the relationship between hybrid WFH and mental distress.

## Methods

### Sample and procedure

A study on hybrid WFH post-pandemic was conducted using "The Working Conditions Survey for Government Agencies" (Norwegian: "Medarbeiderundersøkelsen i Staten"; MUST), a digital work environment survey developed by the National Institute of Occupational Health in Norway (STAMI). The MUST survey is offered to companies of the central government administration in Norway by request from individual organizations and became available in April 2022.

When a company requests to participate, presentations are given by STAMI to inform employees about the general purpose and aims of the survey to ensure they are properly informed that participation is voluntary and that all responses are treated confidentially. The aim of the survey is twofold: (1) for each company, the general psychosocial work environment survey is a practical tool for organizational improvement based on a number of factors previously documented to be of relevance to health, well-being, and productivity. All companies receive standardized reports and a feedback meeting with a representative of STAMI to aid in the interpretation of results. (2) To collect data to research relationships between work and health. To achieve this, STAMI has designed a self-service system for digital work environment surveys, allowing the reliable and secure collection of large volumes of data from a wide range of companies and occupational sectors. For MUST, which is a general psychosocial work environment survey, multiple questionnaire instruments are included to address different research questions.

The MUST survey is a multi-scale work environment survey that compiles a number of several established, validated scales, as well as self-constructed items and scales to comprehensively assess different aspects of the psychosocial work environment. The present study utilized the HSCL-5 to assess mental distress, two items from the General Nordic Questionnaire for Psychological and Social Factors at Work (QPSNordic) (Ørhede et al. 2000), and some self-constructed items to assess hybrid work due to the shortage of established instruments for this purpose prior to the pandemic (see Measurement section below). Since the survey collects information about employees’ health and well-being, thoroughly informing about confidentiality is prioritized to ensure honest responses and satisfy ethical requirements. The study was conducted according to the World Medical Association Declaration of Helsinki and in line with the General Data Protection Regulation (GDPR). STAMI has acquired approval from the Norwegian Centre for Research Data (NSD; approval: 873035).

### Measurements

#### Outcome measures

*Mental Distress* was measured with the five-item version of the Hopkins Symptom Checklist scale (HSCL-5) (Strand et al. 2003), a previously validated self-report instrument to assess symptoms of mental distress (Schmalbach et al. 2021). The HSCL-5 includes five items, and participants report the degree of anxiety and depression symptoms they experienced during the previous week. The items are "Feeling tense or agitated," “Feeling scared or anxious,” “Feeling hopeless about the future,” “Feeling depressed or melancholy,” and “Feeling worried or restless”. Each of the five items had a scale ranging from (1) not at all, (2) a little bit, (3) quite a bit, and (4) extremely. A cutoff score of 2 was used to identify the level of clinically relevant mental distress (Strand et al. 2003; Rodríguez-Barragán et al. 2023), with scores of > 2 indicating higher levels of mental distress and < 2 indicating lower levels of mental distress. The Cronbach’s α coefficient for this scale in the study was 0.85.

### Exposure measure

*Hybrid WFH* was assessed with one item designed to reflect the occurrence of WFH with a fixed or flexible agreement: “Do you sometimes work from home? The options were: (1) No”, “(2) Yes, fixed every week”, and “(3) Yes when needed or desired”.

*The number of days of hybrid work from* home was measured by the item “How many days in a typical week do you work from home?” with response options “One day,” “Two days,” “Three days,” “Four days,” “Five days,” and “6 or more days.”

*Availability demands* were measured by the item: “Are you expected to be available outside of your contracted working hours?”. The scale ranged from “(1) very seldom”, “(2) rather seldom”, “(3) sometimes”, “(4) rather often”, to “(5) very often or always”.

*Work-life conflict* was measured by a single item (Ørhede et al. 2000): “Do the demands of your work interfere with your home and family life?” The scale ranged from “(1) very seldom”, “(2) rather seldom”, “(3) sometimes”, “(4) rather often”, to “(5) very often or always”.

*Life-work conflict* was measured by a single item (Ørhede et al. 2000): “Do the demands of your family or spouse/partner interfere with your work-related activities?”. The scale ranged from “(1) very seldom”, “(2) rather seldom”, “(3) sometimes”, “(4) rather often”, to “(5) very often or always”.

*Workplace control*, or self-determination of WFH, is defined in this study as the degree to which oneself determines whether to work from home. This item was measured by a single item designed to distinguish between degrees of voluntariness: “I work from home because I wish to do so.” The response options were “(1) not at all”, “(2) rarely”, “(3) somewhat”, “(4) to a large degree”, to “(5) to a very large degree”.

### Control variables

The participants’ personal identification number, required to participate in the survey, contained information on gender and age. Each participant provided their own education level (primary, lower secondary, upper secondary, undergraduate, graduate and above). All analyses were adjusted for gender, age, and educational level.

### Response rate

There was a total of 50,879 initially contacted individuals. We received 28,989 answered surveys, resulting in an overall response rate of 57%. Since the research question pertained to hybrid work, we excluded participants without access to an office at the employer’s premises and those with incomplete survey responses for our variables. After these exclusions, our final sample included 24,763 office workers. Thus, out of all the individuals who were initially contacted to participate in the survey, 49% are part of the final sample in this study.

### Statistical analyses

All analyses were conducted using R Statistical Software version 2023.12.0+369 (R Core Team 2020) and Mplus (Muthén and Muthén 1998–2017). The package MplusAutomation (Hallquist and Wiley 2018) was used to run R in Mplus. Data were analyzed by linear and logistic regressions.

## Results

### Descriptive statistics

Table 1 presents descriptive statistics for the sample. Flexible agreement to hybrid WFH was most common (58%), followed by employees who did not have access to hybrid WFH (22%) and, lastly, employees with a fixed agreement (20%). The average score for mental distress from the HSCL-5 was 1.52 [standard deviation (SD 0.6)]. The mean age was 46.4 (SD 11.1) years, and 65 percent of the sample were female.

**Table 1:** Descriptive statistics

Variable	Mean (SD)	N (%)
Working from home		
No		5312 (22)
Yes, fixed every week		4979 (20)
Yes, when needed or desired		14,472 (58)
Age	46.4 (11.1)	
Gender		
Female		16,144 (65)
Male		8619 (35)
Education, mean (SD)	4.4 (0.7)	
(1) Primary		80 (0.3)
(2) Lower secondary		505 (2)
(3) Upper secondary		1722 (7)
(4) Undergraduate		10,291 (42)
(5) Graduate and above		12,165 (49)
Days working from home	1.03 (0.7)	
0		5312 (21)
1		13,455 (54)
2		5817 (24)
3		119 (1)
4		16 (0.1)
5 +		44 (0.2)
Mental distress score	1.52 (0.6)	
< 2		19,674 (79)
> 2		5089 (21)
Life-work conflict	1.6 (0.8)	
(1) Very seldom		13,607 (55.3)
(2) Rather seldom		6991 (28.5)
(3) Sometimes		3310 (13.5)
(4) Rather often		592 (2.4)
(5) Very often or always		68 (0.3)
Missing		195
Availability demands	2.1 (1.1)	
(1) Very seldom		10,458 (42.3)
(2) Rather seldom		5948 (24.1)
(3) Sometimes		5581 (22.6)
(4) Rather often		1828 (7.4)
(5) Very often or always		900 (3.6)
Missing		48
Work-life conflict	2.2 (1.1)	
(1) Very seldom		7336 (29.8)
(2) Rather seldom		7450 (30.3)
(3) Sometimes		6843 (27.8)
(4) Rather often		2511 (10.2)
(5) Very often or always		485 (1.9)
Missing		138
Work-place control	1.4 (0.8)	
(1) To a very large degree		14,531 (75)
(2) To a large extent		3193 (16)
(3) Somewhat		1144 (6)
(4) Rarely		429 (2)
(5) Not at all		154 (1)
Missing		5312

N = 24,763

*SD* standard deviation

### Results from regressions for the complete sample

#### Results from regressions with mental distress as the dependent variable and work factors as the independent variables (Table 2)

Associations between hybrid WFH factors and mental distress for the complete sample (Table 2) showed a statistically significant lower risk of clinically relevant mental distress for employees with access to flexible agreements of hybrid WFH [OR 0.88, 95% CI 0.81–0.976] and with higher workplace Control [OR 0.86, 95% CI 0.82–0.91]. A higher risk of reporting clinically relevant levels of mental distress was found for the increase in the number of days accessing hybrid WFH [OR 1.21, 95% CI 1.12–1.30], availability demands [OR 1.14, 95% CI 1.11–1.18], work-life conflict [OR 2.21, 95% CI 2.13–2.30], and life-work conflict [OR 1.49, 95% CI 1.43–1.56]. We found no statistically significant association between mental distress and fixed agreement of hybrid WFH [OR 1.04, 95% CI 0.93–1.16].

**Table 2:** Results from regressions with mental distress as dependent and work factors as independent

	Mental distress^a^	
	OR	95% CI
Hybrid WFH		
No	Ref	–
Fixed agreement	1.04	[0.93, 1.16]
Flexible agreement	**0.88**	**[0.81, 0.97]**
No. of days hybrid WFH per week	**1.21**	**[1.12, 1.30]**
Availability demands	**1.14**	**[1.11, 1.18]**
Work-life-conflict	**2.21**	**[2.13, 2.30]**
Life-work-conflict	**1.49**	**[1.43, 1.56]**
Work-place control (among those WFH)	**0.86**	**[0.82, 0.91]**

Bold text indicates statistically significant associations

*OR* odds ratio, *CI* confidence interval

^a^Logistic regression with clinically relevant mental distress (HSCL-5) as outcome, adjusted for age, gender, and education

#### Results from regressions with work factors as dependent and hybrid WFH agreements as independent (Table 3)

Table 3 shows a statistically significant negative association between availability demands and having a hybrid fixed work agreement, compared to not working from home [B = − 0.14, 95% CI − 0.18, − 0.10]. However, employees with a fixed agreement also reported higher life-work conflict [B = 0.17, 95% CI 0.14, 0.20].

**Table 3:** Results from regressions with Work factors as dependent and Hybrid WFH agreements as independent (N = 24,763)

	Availability demands		Work-life conflict		Life-work conflict		Work-place control	
Hybrid WFH								
No	Ref	–	Ref	–	Ref	–	–	–
Fixed	**− 0.14**	**[− 0.18, − 0.10]**	0.03	[**− **0.01, 0.07]	**0.17**	**[0.14, 0.20]**	Ref	
Flexible	**0.19**	**[0.15, 0.22]**	**0.14**	**[0.11, 0.18]**	**0.16**	**[0.14, 0.19]**	**− 0.30**	**[− 0.32, − 0.28]**

*B* regression coefficient, *CI* confidence interval

Bold text indicates statistically significant associations

Employees with a flexible agreement report higher availability demands [B = 0.19, 95% CI 0.15, 0.22], higher work-life conflict [B = 0.14, 95% CI 0.11, 0.18], and higher life-work conflict [B = 0.16, 95% CI 0.14, 0.19] than those not working from home. Workplace control for employees with a flexible agreement was lower than that of employees with fixed agreements [B = -0.30, 95% CI − 0.32, − 0.28].

### Results from stratified regressions (by WFH arrangement and by gender)

Separate analyses were conducted to compare the group that reported working from home with a flexible WFH agreement with those that had a fixed WFH arrangement (excluding those not working from home). Thus, it was possible to determine whether the associations between exposures and mental distress were different for the two types of WFH arrangements. Furthermore, additional subgroup analyses were conducted to explore gender differences.

#### Results from subgroup analyses by WFH agreement (fixed or flexible): mental distress as dependent and work factors as independent variables (Table 4)

Employees with a fixed agreement had a significantly higher risk of clinically relevant levels of mental distress associated with availability demands (Table 4) compared to the group with a flexible agreement (fixed agreement OR 1.25, 95% CI 1.16–1.35; flexible agreement OR 1.14, 95% CI 1.09–1.19, p = 0.03). In addition, employees with a flexible agreement had a significantly higher risk of mental distress with an increase in the number of days of Hybrid work per week (flexible agreement OR 1.28, 95% CI [1.16, 1.41], p < 0.01). There were no statistically significant differences between both groups for work-life conflict, life-work conflict, and workplace control.

**Table 4:** Results from subgroup analyses by WFH agreement (fixed or flexible): mental distress is dependent, and work factors are independent variables (N = 24,763); (fixed agreement N = 4979, Flexible agreement N = 14,472)

	Mental distress		Mental distress		Wald test of difference between coefficients^**a**^
	WFH Fixed		WFH Flexible		
	OR	95% CI	OR	95% CI	
No. of Days Hybrid WFH per week	0.93	[0.79, 1.10]	**1.28**	**[1.16, 1.41]**	p < 0.01*
Availability demands	**1.25**	**[1.16, 1.35]**	**1.14**	**[1.09, 1.19]**	p = 0.03*
Work-life-conflict	**2.29**	**[2.11, 2.48]**	**2.23**	**[2.12, 2.35]**	p = 0.59
Life-work-conflict	**1.50**	**[1.38,1.63]**	**1.49**	**[1.41, 1.57]**	p = 0.89
Work-place Control (among those WFH)	**0.81**	**[0.69, 0.95]**	**0.85**	**[0.80, 0.89]**	p = 0.60

Mental distress regressed on independent variables by WFH agreement

Bold text indicates statistically significant associations. Logistic regressions with clinically relevant mental distress (HSCL-5 score > 2) as outcome, adjusted for age, gender, and education

*OR* odds ratio, *CI* confidence interval

^a^Wald test of difference of coefficients between subgroups

#### Results from subgroup analyses by gender: mental distress as dependent variable and work factors as independent (Table 5)

Subgroup analyses by gender show a statistically significant difference in the risk of clinically relevant levels of mental distress associated with availability demands, with men at significantly higher risk than women (women OR 1.11, 95% CI, 1.07–1.16; men OR 1.21, 95% CI 1.14–1.27, p = 0.02) (see Table 5).

**Table 5:** Results from subgroup analyses by gender: mental distress as dependent variable and work factors as independent (N = 24,763) (female = 16,144, men = 8,619)

	Mental distress		Mental distress		Wald test^a^
	Female		Male		
	OR	95% CI	OR	95% CI	
Hybrid WFH					
No	Ref	–	Ref	–	–
Fixed agreement	1.10	[0.96, 1.25]	0.91	[0.74, 1.12]	p = 0.14
Flexible agreement	0.90	[0.81, 1.01]	**0.84**	**[0.72, 0.98]**	p = 0.46
No. of days hybrid WFH per week	**1.21**	**[1.11, 1.32]**	**1.20**	**[1.05, 1.37]**	p = 0.95
Availability demands	**1.11**	**[1.07, 1.16]**	**1.21**	**[1.14, 1.27]**	p = 0.02*
Work-life-conflict	**2.20**	**[2.10, 2.30]**	**2.23**	**[2.09, 2.38]**	p = 0.75
Life-work conflict	**1.49**	**[1.42, 1.57]**	**1.50**	**[1.39, 1.61]**	p = 0.98
Work-place control (among those WFH)	**0.81**	**[0.69, 0.95]**	**0.85**	**[0.80, 0.89]**	p = 0.10

Mental distress regressed on independent variables. OR = Odds ratio

Bold text indicates statistically significant associations. Logistic regressions with clinically relevant mental distress (HSCL-5 score > 2) as outcome, adjusted for age, gender, and education

*CI* confidence interval

^a^Wald test of difference of coefficients between subgroups

#### Results from subgroup analyses by gender: results from regressions with work factors as dependent and hybrid WFH agreements as independent (Supplementary Table 2)

Subgroup analyses by gender show a statistically significant difference between women and men regarding workplace control, where women with a flexible agreement report lower workplace control compared to men (women: B = − 0.35; 95% CI − 0.38, − 0.31; men: − 0.28 95% CI − 0.30, − 0.26; p < 0.01) (see Supplementary Table 2).

## Discussion

The present study revealed that having access to flexible hybrid WFH and having workplace control were associated with a lower risk of clinically relevant mental distress compared to not having access to hybrid work or working from home with a fixed agreement. These findings corroborate previous research that highlights positive associations between autonomy to self-chose hybrid work and mental health (Moring 2022). Nevertheless, while flexible work arrangement was associated with better mental health in the total sample, it seems likely that there are several important potential pitfalls and boundary conditions that should guide the use of such working arrangements. That is, flexible WFH was associated with availability demands, work-life- and life-work conflict, and was less likely to be self-chosen than fixed arrangements. Previous reviews have suggested that the effect of WFH depends on conditions such as whether it is self-determined or mandatory (Dalsbø et al. 2024; Fløvik et al. 2021), and our results strengthen the notion that hybrid work exhibits a complex, dynamic relationship with well-being.

It is important to note that the cross-sectional nature of the data precludes strong conclusions about causality or development over time—many alternative explanations to the interpretations in this study exist and cannot be ruled out. For instance, the observed inverse relationship between flexible work arrangements and mental distress could reflect a selection of individuals who are not yet burdened by mental health issues due to availability demands and work-life conflict and, therefore, more capable of WFH without strain. Further research should investigate potential dynamics longitudinally to provide more nuanced insights.

The expectation for employees to be available outside regular hours to meet the organization’s demands while working from home has been linked to adverse health factors, including difficulties detaching from work once the workday is over and emotional exhaustion (Dettmers 2017; Lutz et al. 2020; Dettmers et al. 2016). This constant availability has been also associated with negative work factors, such as lower organizational commitment, intention to leave (Knardahl and Christensen 2022), and work-life conflict (Lutz et al. 2020; Brauner et al. 2022). Our study contributes to these findings by showing that flexible hybrid work arrangements were associated with higher availability demands and greater life-work conflict, whereas fixed agreements were linked to lower availability demands and not associated with work-life conflict. Although flexible agreements may offer higher job control, they may blur the lines between working hours and personal life, leading to mental distress. Not surprisingly, we also observed that mental distress was more common with higher availability demands, work-life conflict and life-work conflict.

In subgroup analyses, employees with fixed agreements exhibited a stronger negative response to availability demands compared to those with a flexible agreement. This discrepancy may be due to employees with a fixed agreement expecting to be contacted only during designated hours. Conversely, employees with a flexible agreement might have a different attitude to being contacted at any given time, reflecting their non-specific remote-working schedule. Alternatively, this could be due to pre-existing differences between the groups, such as more vulnerable workers selected into having a fixed arrangement. Nonetheless, as mentioned earlier, the group with a fixed agreement did not exhibit an elevated risk of mental distress, suggesting that availability demands are particularly stressful when one has a set arrangement to work from home.

These patterns, while appearing somewhat inconsistent, highlight the nuances of the “flexibility paradox” (Chung 2022). On the one hand, flexible work arrangements may help employees manage work challenges more effectively, potentially reducing sources of rumination and increasing problem-focused coping. On the other hand, digital WFH can lead to feelings of being “always on” and blur the boundaries between work and personal spaces, also called techno-invasion (Tarafdar et al. 2010). Our study aligns with previous research indicating that both flexible and fixed agreements are associated with higher levels of life-work conflict (Chung 2022; Mazmanian et al. 2013). This underscores the complexities of WFH, regardless of the reason or arrangement, and suggests the need to consider specific configurations, such as having a dedicated space for WFH or cohabitants who also WFH.

Interestingly, employees with a flexible agreement reported a particularly higher risk of work-life conflict compared to counterparts with a fixed agreement. This could possibly point to flexible arrangements more often being supplemental rather than replacing work at the employer’s premises. If so, this could extend the total workload, and the arrangement, though providing flexibility, may paradoxically contribute to work-life conflict due to the inability to fully disconnect from work (Chung 2022; Mazmanian et al. 2013). Employers have a critical role in establishing strategies that allow clear boundaries to support work-life balance across the organization.

The group with a flexible agreement had significantly higher odds of reporting mental distress with an increase in the number of days of hybrid work per week. A recent study found that office workers who worked from home two days a week reported higher job satisfaction and less feelings of isolation, while working more days from home could pose a risk for mental distress (Choudhury et al. 2024). This suggests that unregulated and flexible agreements might lead to isolation from the work environment, thereby contributing to mental distress.

Our results ought to provide recommendations to practitioners about how to manage the “new normal” of hybrid work in such a way that it supports employees’ mental health. Allowing employees the flexibility to work from home when needed may be helpful, as it may provide a sense of autonomy and enhance control over work execution, promoting mastery and preventing distress. However, there are clear indications that “too much” flexibility may counteract these benefits. WFH may instigate availability demands and work-life conflict or may be symptomatic of attempts to compensate for excessive workloads. Thus, organizations can provide mental health support by actively promoting work-life balance and establishing norms and guidelines that discourage excessive WFH. It is important to distinguish between allowing access to WFH and expecting employees to WFH. Such guidelines may include how to use communication technologies while WFH, which may reduce availability demands outside of work hours.

Although Norway is considered a country with a high degree of gender equality (Nilsen et al. 2017) our study observed notable patterns in mental distress outcomes with regard to WFH and gender differences. Women did not show the same beneficial association of flexible hybrid WFH with less mental distress as men. This could be in agreement with “the double burden hypothesis”, suggesting that the beneficial effects of hybrid WFH are offset by women’s additional responsibilities at home. Interestingly, however, there was no evidence to suggest that women experienced more availability demands, work-life conflict, or life-work conflict than men when performing hybrid WFH. Moreover, the association of availability demands with mental distress was significantly weaker for women, suggesting women may be more resilient to such demands. On the other hand, there were indications that a flexible arrangement, as opposed to fixed, were less often a self-chosen practice for women compared to men. While the cross-sectional design limits definitive conclusions on the double burden hypothesis, these findings raise important questions about gender roles in hybrid work environments. Further research on gender differences should examine work-family conflict measurements prospectively to understand the long-term effects of how flexible arrangements truly impact women and men. This is to have an inclusive approach when proposing policies around flexible work (Chung 2024) that improve employees’ work-life and life-work balance, reduce mental distress, and support gender equality.

### Limitations and future research

This study is one of few large-scale post-pandemic studies investigating hybrid WFH in relation to mental distress and work factors relevant to mental distress. Our findings offer several insights relevant to policymakers and organizational practitioners. Nevertheless, some limitations must be acknowledged and kept in mind when interpreting the results. First, important observations can be made about what characterizes those who practice hybrid work, but causality cannot be inferred from cross-sectional data. Further studies should explore possible mechanisms by which flexible work agreements can improve employees' mental health by acquiring longitudinal data, conducting interventions, and incorporating different measurement methods. Note that cross-sectional data precludes studying the temporal order of variables, detecting changes in predictors and outcomes, as well as studying cumulative exposure to determine long-term implications. Another central limitation was the use of single items to measure central constructs. This reduces precision and is a potential validity threat. However, for some constructs, single items could be more reasonable, such as reporting the number of days one has worked from home. Nevertheless, future studies should aim to include more comprehensive measures. Finally, our sample included participants from the public sector, which limits external validity and our findings' applicability to office workers from other sectors.

## Concluding remarks

Our study draws attention to the complexities and mental health implications posed by hybrid work arrangements, emphasizing the need for attention to policies that support employees’ mental health. Future research should focus on longitudinal analyses to understand the nuanced factors of flexible and fixed work arrangements on mental health across diverse workforce demographics.

## Supplementary Information

Below is the link to the electronic supplementary material.

Supplementary file1 (DOCX 25 KB)

## Data Availability

The data that support the findings of this study are available from the corresponding author upon reasonable request.
