# Supplementary material for: Hybrid work and mental distress: a cross-sectional study of 24,763 office workers in the Norwegian public sector
Source: Int Arch Occup Environ Health. 2025 Mar 25;98(4-5):399–407. doi: 10.1007/s00420-025-02136-9 (PMC12238147; doi:10.1007/s00420-025-02136-9)
Supplement: Supplementary file 1 — Supplementary file1 (DOCX 25 KB) [file 420_2025_2136_MOESM1_ESM.docx]

**Supplementary Tables**

**Supplementary table 1. Descriptive statistics for Women and Men. [SD = standard deviation], (Female= 16,144, Men = 8,619).**

|  | Women (N=16,144) | | Men (N=8,619) | |  |
| --- | --- | --- | --- | --- | --- |
| Variable | Mean (SD) | N (%) | Mean (SD) | N (%) |  |
| **Working from Home** |  |  |  |  |  |
| No |  | 3262 (20) |  | 2050 (24) |  |
| Yes, fixed every week |  | 3571 (22) |  | 1408 (16) |  |
| Yes, when needed or desired |  | 9311 (58) |  | 5161 (60) |  |
| **Age** | 45.7 (10.9) |  | 47.5 (11.3) |  |  |
| **Education, mean (SD)** | 4.4 (0.7) |  | 4.4 (0.9) |  |  |
| 1. Primary |  | 43 (0.2) |  | 37 (0.4) |  |
| 1. Lower Secondary |  | 329 (2.0) |  | 176 (2.0) |  |
| 1. Upper Secondary |  | 1071 (6.6) |  | 651 (7.55) |  |
| 1. Undergraduate |  | 6934 (43.0) |  | 3357 (38.9) |  |
| 1. Graduate and above |  | 7767 (48.1) |  | 4398 (51.0) |  |
| **Days Working from home** | 1.0 (0.7) |  | 1.0 (0.7) |  |  |
| 0 |  | 3262 (20.2) |  | 2050 (23.8) |  |
| 1 |  | 8754 (54.2) |  | 4701 (54.5) |  |
| 2 |  | 4022 (25.0) |  | 1795 (20.8) | |
| 3 |  | 72 (0.4) |  | 47 (0.55) |  |
| 4 |  | 9 (0.1) |  | 7 (0.08) |  |
| 5+ |  | 25 (0.2) |  | 19 (0.22) |  |
| **Mental Distress Score** | 1.5 (0.6) |  | 1.5 (0.6) |  |  |
| <2 |  | 12626 (78.2) |  | 7048 (81.8) |  |
| >2 |  | 3518 (21.8) |  | 1571 (18.2) |  |
| **Life-Work Conflict** | 1.6 (0.8) |  | 1.7 (0.8) |  |  |
| 1. Very seldom |  | 9122 (57.0) |  | 4485 (52.4) |  |
| 1. Rather seldom |  | 4395 (27.5) |  | 2596 (30.3) |  |
| 1. Sometimes |  | 2103 (13.1) |  | 1207 (14.1) |  |
| 1. Rather often |  | 353 (2.2) |  | 239 (2.8) |  |
| 1. Very often or always |  | 37 (0.2) |  | 31 (0.4) |  |
| Missing |  | 134 |  | 61 |  |
| **Availability Demands** | 1.9 (1.1) |  | 2.3 (1.2) |  |  |
| 1. Very seldom |  | 7524 (46.7) |  | 2934 (34.1) |  |
| 1. Rather seldom |  | 3768 (23.3) |  | 2180 (25.3) |  |
| 1. Sometimes |  | 3332 (20.6) |  | 2249 (26.1) |  |
| 1. Rather often |  | 1005 (6.2) |  | 823 (9.6) |  |
| 1. Very often or always |  | 478 (2.9) |  | 422 (4.9) |  |
| Missing |  | 37 |  | 11 |  |
| **Work-Life Conflict** | 2.3 (1.1) |  | 2.2 (1.0) |  |  |
| 1. Very seldom |  | 4726 (29.5) |  | 2610 (30.4) |  |
| 1. Rather seldom |  | 4663 (29.1) |  | 2787 (32.5) |  |
| 1. Sometimes |  | 4606 (28.7) |  | 2237 (26.1) |  |
| 1. Rather often |  | 1720 (10.7) |  | 791 (9.2) |  |
| 1. Very often or always |  | 336 (2.1) |  | 149 (1.7) |  |
| Missing |  | 93 |  | 45 |  |
| **Life-Work Conflict** | 1.6 (0.8) |  | 1.7 (0.8) |  |  |
| 1. Very seldom |  | 9122 (57.0) |  | 4485 (52.4) |  |
| 1. Rather seldom |  | 4395 (27.5) |  | 2596 (30.3) |  |
| 1. Sometimes |  | 2103 (13.1) |  | 1207 (14.1) |  |
| 1. Rather often |  | 353 (2.2) |  | 239 (2.8) |  |
| 1. Very often or always |  | 37 (0.2) |  | 31 (0.4) |  |
| Missing |  | 134 |  | 61 |  |
| **Work-place Control** | 4.7 (0.7) |  | 4.5 (0.9) |  | |
| (1) Not at all |  | 83 (0.6) |  | 71 (1.1) | |
| (2) Rarely |  | 222 (1.7) |  | 207 (3.1) | |
| (3) Somewhat |  | 614 (4.8) |  | 530 (8.1) | |
| (4) To a large extent |  | 1834 (14.2) |  | 1359 (20.7) | |
| (5) To a very large degree |  | 10129 (78.6) |  | 4402 (67.0) | |
| Missing |  | 3262 |  | 2050 | |

**Supplementary table 2. Results from subgroup analyses by gender: Work factors as dependent variable and type of Work agreement as independent (N=24763).**

|  | Availability Demands  B  95% CI | | | Work-Life Conflict  B  95% CI | | | Life-Work conflict  B  95% CI | | | Work-place Control  B  95% CI | | |
| --- | --- | --- | --- | --- | --- | --- | --- | --- | --- | --- | --- | --- |
|  | Female | Male | Wald Test**^a^** | Female | Male | Wald Test**^a^** | Female | Male | Wald Test**^a^** | Female | Male | Wald Test**^a^** |
| No | Ref | Ref |  | Ref | Ref |  | Ref | Ref |  |  |  |  |
| Fixed agreement of Hybrid WFH | **-0.14**  **[-0.19, -0.09]** | **-0.16**  **[-0.23, -0.08]** | p = 0.69 | 0.03  [-0.02, 0.08] | 0.02  [-0.05, 0.09] | p = 0.75 | **0.17**  **[0.13, 0.21]** | **0.17**  **[0.12, 0.23]** | p = 0.87 | Ref | Ref |  |
| Flexible agreement of Hybrid WFH | **0.17**  **[0.13, 0.22]** | **0.21**  **[0.15, 0.27]** | p = 0.31 | **0.15**  **[0.10, 0.19]** | **0.14**  **[0.09, 0.19]** | p = 0.75 | **0.16**  **[0.13, 0.19]** | **0.17**  **[0.12, 0.21]** | p = 0.79 | **-0.35**  **[-0.38, -0.31]** | **-0.28**  **[-0.30, -0.26]** | **p< 0.01*** |

Note: Note: B = Regression Coefficient; CI = Confidence interval. **Bold text** indicates statistically significant associations. Linear regressions with work factors as outcome based on subgroups by gender, adjusted for age and education. ^a^ Wald test of difference of coefficients between subgroups.
